# A Cross-Sectional Study Comparing Younger and Older Nursing Home Residents in Western Canada

**DOI:** 10.1093/geroni/igab046.1591

**Published:** 2021-12-17

**Authors:** Bianca Shieu, Todd Schwartz, Anna Beeber, Matthias Hoben, Mark Toles, Ruth Anderson

**Affiliations:** 1 University of Pittsburgh/ School of Medicine, Pittsburgh, Pennsylvania, United States; 2 UNC Chapel Hill, UNC Chapel Hill, North Carolina, United States; 3 University of North Carolina at Chapel Hill, UNC Chapel Hill, North Carolina, United States; 4 University of Alberta at Edmonton, Edmonton, Alberta, Canada

## Abstract

Specialized care for younger nursing home (NH) residents may be necessary to meet their unique health and quality of life needs; however, key attributes of younger NH residents are poorly understood and limit the development of effective, tailored interventions. This study described differences in clinical and nonclinical characteristics of younger vs. older nursing NH residents. In a retrospective cohort study, we used SPSS and analyzed comprehensive Resident Assessment Instrument – Minimum Data Set (RAI-MDS 2.0) data from NHs in Western Canada, for the period from January 2016 to December 2017. We included all assessments (full and abbreviated) performed quarterly. These findings indicated that younger (age 18-64) vs. older (age >=65) NH residents differed considerably: younger residents were predominately male, single, more obese, more depressed, had higher prevalence of depression, cerebral vascular accident, and hemi- or quadriplegia, and required more assistance in activities of daily living than older residents. The findings will contribute a better comprehension of the characteristics of the younger NH population and how they differ from other residents. The study provides useful information to policymakers, providers, and researchers to guide them in developing tailored policies, programs, and interventions. Also, findings may guide consumers as they plan for long-term care needs of loved ones. Finally, the findings provide a baseline estimate as researchers continue to track the growth of and changes in, the populations served in nursing homes.

